# Integrative Western and Chinese Medicine on Coronary Heart Disease: Where Is the Orientation?

**DOI:** 10.1155/2013/459264

**Published:** 2013-08-19

**Authors:** Siming Li, Hao Xu

**Affiliations:** ^1^Graduate School, Beijing University of Chinese Medicine, Beijing 100029, China; ^2^Cardiovascular Diseases Center, Xiyuan Hospital, China Academy of Chinese Medical Sciences, Beijing 100091, China

## Abstract

Coronary heart disease (CHD) is the leading cause of death. As the main treatment of CHD, modern medicine has improved dramatically in recent years. Although researches of TCM and integrative medicine on CHD are witnessed encouraging progress in many respects, the role TCM playing in the prevention and treatment of CHD has been unprecedentedly challenged under such circumstance of the very fast development of modern medicine. In order to share mutual complementary advantages of TCM and western medicine, this review summarizes the relatively prominent researches of TCM and integrative medicine on CHD in recent years, and illuminates the issue of the orientation of the further research of integrative medicine on CHD, including (1) original innovation of TCM etiology and pathogenesis, (2) combination of disease and TCM syndrome, (3) biological basis of TCM syndrome of CHD, (4) clinical design and quality control of integrative medicine research, (5) herb-drug interaction, (6) difficulties and hot issues of modern medicine.

## 1. Strengthening Original Innovation on the TCM Etiology and Pathogenesis of CHD

So far, there are so many researches of TCM and integrative medicine on CHD, however, with insufficient enhancement of clinical effectiveness. If the work on blood-stasis syndrome (BSS) is a milestone of TCM and integrative medical research on CHD, activating blood circulation by removing blood-stasis and its derived therapies have significantly improved the clinical effectiveness of TCM therapy on CHD [1], when comparing with the traditional therapeutic method of eliminating stagnation to activate yang mainly with formulae on the basis of Trichosanthes and Allium. After the research of BSS, it is urgent to strengthen the original innovation in the TCM and integrative medicine research. Focusing on the clinical problems or phenomenon, we should innovate TCM etiology and pathogenesis of CHD, which will bring about innovation on the treatment of CHD and then improve the clinical effectiveness. In this respect, the etiology and pathogenesis innovation research on “blood-stasis and toxin” conducted by the research team headed by academician Chen Ke-ji can be used for reference. Directing at the clinical problem/phenomenon that “some patients with CHD have a stable condition for a long time while some develop acute cardiovascular events (ACEs),” the research team innovatively proposed the hypothesis of “blood-stasis and toxin causing catastrophe” on etiology and pathogenesis of CHD [2, 3], according to the modern medicine progress in the relevance of the vulnerable plaque rupture, the inflammatory response of atherosclerosis and acute coronary syndrome (ACS), and on the basis of the previous experimental research [4, 5], long-period clinical practice, as well as the TCM cognition of “toxin”. On that basis, they conducted a series of research combined literature reviews with experimental and clinical researches [6–8], which provides a model for TCM etiological innovation research. Especially, the prospective cohort study [9] enrolling 1503 stable patient with CHD designed by reference to the classical method of clinical epidemiological etiological research analyzed correlation factors of ACEs in the follow-up, proved the hypothesis of “blood-stasis and toxin causing catastrophe,” and established the criterion of syndrome differentiation of “toxin” for stable CHD. It is of great significance for exerting TCM features and advantages of “preventive treatment” to early recognize high-risk patients and give intervention as soon as possible, and further reducing the incidence of ACEs. In addition, some scholars proposed the hypothesis of “endogenous collateral wind” in accordance with the characteristic of the acute onset and rapid progression of ACS, which is also a beneficial exploration [10]. All in all, in view of the different group of patients with CHD, to conduct further research on their pathological physiology proceeding from the similarities and differences in the theory of TCM and Western medicine and then to analyze combining with their characteristics of TCM etiology, pathogenesis, and syndrome, we may bring about the original innovation and the development of TCM therapy to further improve the clinical effectiveness.

## 2. Combination of Disease and TCM Syndrome: One of the Breakthrough Points of Integrative Medicine Research on CHD

 Combining disease with TCM syndrome is an important treatment model in TCM clinical practice. The theory of combining disease with syndrome reflects the principle of inheritance and innovation, which has been widely accepted by TCM and integrative medical practitioners for the advantages of scientificity and operability. Thus, it is the important breakthrough point of integrative medicine research on CHD. Previously, most of the TCM syndrome researches on CHD are cross-sectional studies focusing on syndrome distribution characteristics of CHD and different subgroups, which have made certain progress. Many results have shown that patients with CHD have the syndrome of asthenia in origin and asthenia in superficiality, and blood-stasis is the main syndrome element of CHD, followed by qi deficiency [11, 12]. There are also different characteristics in each subtyps: for instance, the TCM syndrome of exertional angina is primarily qi deficiency [13]; the syndrome distribution of AMI is mostly identical to which of CHD but more serious of both asthenia in origin and asthenia in superficiality [14, 15]. In recent years, characteristics of TCM syndrome distribution of CHD, such as increasing heat-accumulation [16], aggravating qi deficiency after coronary revascularization [17] are worthy of attention. The characteristics of syndrome distributions in patients with CHD combined with different diseases are shown in difference: for example, phlegm-dampness and blood-stasis syndrome may be the main characteristic of CHD combined with hyperlipidemia [18, 19]; when CHD combined with hypertension, BSS is manifested more obvious, while liver fire and hyperactivity of yang are also the common syndrome elements [20, 21]; when combined with diabetes mellitus (DM), BSS and turbid phlegm are common in excessive syndrome while deficiency of both qi and yin is most common in deficiency syndrome [20, 22]. A regional difference also exists in the TCM patterns of patients with CHD between the North and South China: the proportions of patients with qi-deficiency syndrome, turbid phlegm syndrome, or blood-stasis syndrome were generally higher in the South group, while the proportion of patients with a congealing cold syndrome was identified to be obviously higher in the North group [23]. The research on diagnostic criteria of combining disease with syndrome is also going deeper, such as the establishment of the diagnostic criteria for CHD patients with BSS [24], which is the further research based on the diagnostic criteria of BSS and is of great significance in standardizing syndrome research and improving the level of TCM syndrome differentiation and treatment.

 It is worth mentioning that in the process of the occurrence, development, and prognosis of CHD, TCM syndrome is always in a dynamic change, so the research on the evolution law of CHD syndromes may help us understand the pathogenesis and prognosis of CHD in each stage or population and then improve clinical syndrome differentiation and treatment. But research in this field is still less and need to be strengthened. Mei et al. [25] conducted a case-control study to explore the main factors and the influencing extent of the susceptibility of the Han population with CHD of BSS in Fuzhou area. The result showed that mental labors, hypertension, excessive consumption of oil and salt, depression, stress, and past relevant medical history are related to the predisposing factors for the Han population with CHD of BSS in Fuzhou area when compared with the population of non-BSS. It has provided clues for the cause of CHD of BSS. Through method of principal component and logistic regression analysis, we studied the distribution laws of TCM syndrome elements in 1072 CHD inpatients according to the 1-year follow-up of cardiovascular events [26]. The results showed that blood-stasis, qi deficiency, turbid phlegm, and yin deficiency were the main syndrome elements of CHD inpatients while qi deficiency and yin deficiency might be relevant TCM syndrome elements of the CHD in patients who suffered from follow-up ACEs. In addition, we applied multifactor dimensional reduction (MDR) and complex network method to analyze the evolution law of TCM syndrome in 1333 stable patients with CHD [27]. The result showed that with the time of follow-up, the TCM syndromes were kept in a dynamic evolution. Toxin resulting from blood-stasis, combination of toxin and blood-stasis, toxin consuming qi, and blood-stasis due to qi deficiency may be the key pathogenic mechanism and the law of syndrome development.

## 3. Research on Biological Basis of TCM Syndrome of CHD under the Guidance of Holistic Concept

Treatment based on syndrome differentiation is one of the characteristics and advantages of TCM in preventing and treating disease, but how to conduct syndrome differentiation exactly is the key to improve the clinical efficacy. The rapid development of modern medicine has provided technical support and good opportunity for the expansion and extension of the four methods of diagnosis of TCM and the organic combination of traditional macroscopic and modern microcosmic syndrome differentiation. In recent years, Chinese scholars have studied relevant factors affecting TCM syndrome differentiation of CHD and the biological basis of syndrome in the aspects of coronary artery lesion, cardiac function, changes in ECG, blood lipid, insulin resistance, homocysteine and inflammation factors, related gene, and so on [28]. They have made significant progresses and provided the basis for objectifying the TCM syndrome differentiation for CHD. In particular for some silent myocardial ischemia, adopting the method of microcosmic syndrome differentiation can make the treatment more targeted. However, indicators in most of these studies are single and just reflect one aspect of TCM syndrome's essential. The research on biological basis of TCM syndrome should be under the guidance of holistic view and system biology and by taking full advantage of modern omics technologies such as genomics, proteomics, and metabolomics. Researches on this respect have been launched and show favorable signs. For example, applying oligonucleotide microarray technology, through comparing the gene expression profiles among healthy control group, blood-stasis syndrome with CHD and without CHD group, Ma et al. [29] screened the differential genes correlated with CHD of BSS, analyzed gene ontology and pathway, and then confirmed the target gene by real-time reverse transcription polymerase chain reaction (RT-PCR). The results suggested that inflammation and immune response might cause the occurrence and development of blood-stasis syndrome to some extent. Yuan et al. [30] also found that the hereditary relevant differential genes of BSS of CHD were closely associated with inflammation, plaque formation, and endothelial injury by gene chip technique. Wu et al. [31] applied proteomic technology including two-dimensional electrophoresis (2DE), image analysis, and spectrometry detection to identify the change of plasma protein in healthy person and BSS patients with CHD and found that fibrinogen and granzyme might be considered as diagnostic biomarkers of BSS patients with CHD. Li et al. [32] screened out 13 differential proteins from platelet between BSS patients with CHD and healthy control group by proteomic technology, among which 7 were identified by spectrometry successfully. The results showed that CD41 and Actin*γ* are the possible marker proteins that might play crucial roles in the occurrence and development of BSS patients with CHD. Zhao et al. [33] also applied proteomic technology and discovered that unstable angina-qi deficiency and blood-stasis syndrome (UA-QDBS) might belong to a kind of inflammatory reaction. There might be myocardial injury, abnormality of coagulation factor, lipid metabolic disorder, and oxygen transport obstacle in patients of UA-QDBS. Lu et al. [34] proposed a new approach of studying the biological basis of phlegm and blood-stasis syndrome in CHD based on metabonomics and correspondence of prescription and syndrome. Utilizing metabonomics, Jian et al. [35] found that the major plasmic metabolites in patients with CHD- blood-stasis syndrome (CHD-BSS) are arachidonic acid, octadecanoic acid, lactic acid, urea, citric acid, *β*-hydroxybutyric acid, oleic acid, glucose, and alanine. The results showed that CHD-BSS is related with lipid metabolism, glycometabolism, as well as the stress induced by hypoxia and agonia. Applying various omics technologies, we can conduct researches in the holistic level, and provide strong technical support for the research on biological basis of TCM syndrome. 

## 4. Strengthening the Clinical Design and Quality Control of Integrative Medicine Research on CHD

 In recent years, with the concept of evidence-based medicine (EBM) widely accepted, a number of multicenter, randomized controlled trials (RCT) with large sample focusing on the prevention and treatment of CHD have been carried out successively both in the fields of Chinese medicine and integrative medicine, for example, the randomized, double-blind, placebo-controlled trial on the effect of Xuezhikang (XZK) for regulating blood lipids and secondary prevention of CHD [36]. The results showed that XZK-treated group decreased the recurrence of nonfatal myocardial infarction in patients with CHD by 62% compared with the placebo control group, and the occurrence of coronary death, coronary events, and total mortality was reduced by 45%, 30%, and 33.0%, respectively, which fills the blank of research on regulating blood lipids for secondary prevention of CHD in oriental populations. According to restenosis after percutaneous coronary intervention (PCI), a worldwide problem in the field of heart disease, the effectiveness and safety of XS0601 treatment based on conventional Western medicine have been demonstrated in a multicenter, randomized, double-blind, placebo-controlled trial [37, 38]. These trails provided objective evidence for integrative medicine in the prevention and treatment of CHD. Admittedly, RCT has a higher level of evidence than many other trials and is more persuasive. However, we should also avoid only-RCT-oriented clinical trials in clinical research.

 When conducting clinical trials of integrative medicine on CHD, we should pay attention to the following three points.

1. Strengthen the top-level design of clinical trials and choose appropriate clinical research methods based on the clinical demands and research objectives. For most intervention studies, RCT including explanatory randomized controlled trial (ERCT) and pragmatic randomized controlled trial (PRCT) is usually the first choice. Recently, real-world study (RWS) [39], which is applicable to explore the effectiveness of complex intervention with integrative medicine [40] and the reevaluation of postmarketing drugs, has been drawn and is worthy of more attention, while researchers need to strengthen the control of confounding factors, to which modern statistical analysis methods involving instrumental variable and propensity scores can be introduced.
2. Outcome measures should be appropriate [41]. Avoid merely using subjective outcome measures like angina score and TCM syndrome score. On the contrary, objective outcome measures should be adopted, such as electrocardiogram treadmill exercise test on stable angina, and if possible, long-term follow-up of ACEs, to improve persuasion of evidence. Meanwhile, we should pay attention to the quality of life, patient reported outcomes (PRO), and medical economics evaluation to highlight the characteristics of TCM and the advantage of integrative medicine.
3. Trials are designed and conducted according to good clinical practice (GCP) principle. Strengthen the implementation of process management and quality control of clinical trials, such as the international registration of clinical trials, ethical approval, the third-party evaluation of end point, and data management, and reported trials in accordance with the consolidated standards of reporting trials (CONSORT) statement 2010 [42], in order to improve the quality of original trials as source references of systematic reviews (SRs) and meta-analysis [43]. Combining the problems often existing in current clinical trial reports, the following details should be highlighted: estimation of sample size, description of random method and random allocation concealment, patient compliance and the completion status of follow-up, adverse drug reactions and its treatment, data processing of loss to follow-up (LFU) and intention to treat analysis (ITT) or not, and so forth.

## 5. Herb-Drug Interaction Is a Key Problem That Cannot Be Ignored in Integrative Medicine Research on CHD

If the combination of disease and syndrome is the integration of TCM and Western medicine in diagnostic level, then interaction of Chinese and Western medicine can be the practical problem we face in treating disease with integrative medicine. As the integrative medical model of patient-centered healthcare and combined application of botanical and chemical drugs evolving into a new trend of modern medicine in preventing and treating disease nowadays, the combined application of Chinese and Western medicine is increasing, and the potential interactions are drawing more and more attention [44].

Herb-drug interaction has two meanings. One is the advantageous function of effect-enhancing and toxicity-reducing. CHD and other chronic noncommunicable diseases are often related to multiple risk factors, complex interventions, and multitarget treatment are more appropriate. Combination therapy with a TCM formula has been the feature and advantage of TCM. Some international scholars also proposed the concept of “polypill” [45], which has caused widespread concern. Actually antihypertensive polypill has been widely accepted in the treatment of hypertension. Xuezhikang, a modern Chinese patent medicine, which has beneficial effects in lipid regulation and secondary prevention of CHD, could also be regarded as a natural polypill [46], and its multicomponent synergy may be the mechanism of pleiotropic effects. According to our clinical practice experience, the effectiveness of prescription for supplementing qi and activating blood circulation in combination with vasodilator agent and diuretics for patients with heart failure (HF) after AMI is much better than Western medicine only, which suggests the potential synergistic effect of Chinese and Western medicine.

Another meaning of herb-drug interaction is the potential of increased risk of adverse reactions. International researches discovered that some botanical drug products including Chinese herbal medicine, such as *hypericum perforatum*, motherwort, ginseng, gingko biloba, salvia miltiorrhiza, garlic, and aconite, can possibly increase the risk of adverse events in patients with cardiovascular diseases by the interactions with other drugs, particularly in elderly patients who always consume multiple prescription medications in the same time for comorbidities [47]. In clinical practice, it was also found easily to cause digitalis toxicity when taken digitalis with semen lepidii, north acanthopanax bark, and so forth. In addition, patients after PCI are treated with dual antiplatelet therapy with aspirin and clopidogrel. Whether it increases the risk of bleeding or not when adding blood-invigorating and stasis-removing herbs needs to be further investigated.

So, herb-drug interaction is the issue that cannot be ignored in the research of integrative medicine on CHD. In clinical practice, doctors should notice the reference application of evidence-based medication [48, 49], pay great attention to adverse drug interaction that has been discovered, and avoid the off-label drug use, long-term drug overdosage and irrational drug combination, and so forth, so as to minimize the risk of adverse events. To the key population, more attention should be paid to strengthen monitoring and discovering the potential adverse effects of drug interactions timely.

 Meanwhile, the firsthand material should be accumulated in routine clinical practice, as there is an important way to discover the meaningful clues of herb-drug interactions through the real-world data analysis. On this basis, design experiment scientifically, carry out the relevant studies involved in drug metabolism, pharmacokinetic, efficacy, toxicology, and the relevance of toxicity and efficacy, and conduct in-depth study of herb-drug interactions and their mechanism from drug absorption, distribution, transformation, metabolism, excretion, and so on.

## 6. Target Integrative Medicine Research on Difficulties and Hot Issues of Modern Medicine

The development of modern medicine has brought new hope for the prevention and treatment of CHD. However, some new problems have unavoidably been presented, such as aspirin resistance (AR), restenosis after PCI, late thrombosis of drug-eluting stent (DES), the no-reflow phenomenon after revascularization, vulnerable plaque, contradiction of therapeutic angiogenesis, the viability and differentiation ability of transplanted cells when operating stem cell transplantation, and residual cardiovascular risk. These are still challenges which modern medicine has to face positively. Focusing on these key issues that influence the curative effect, we should give full play to the characteristics of TCM, complement each other's advantages of Chinese and Western medicine, and conduct researches scientifically.

For instance, to the clinical problem of AR, we may screen potential Chinese herbals which have antiplatelet effect, then explore its material basis and active ingredients, illustrate the target point of drug action, find the lead compound, and optimize its structure. An effective Chinese herb with high-efficacy and low-toxicity will be of great significance in the prevention and treatment of cardiovascular disease. Some domestic scholars [50] have recomposed Ferulic Acid and Ligustrazine to give full play to the advantages of Chinese compound prescription and observed its inhibitory effect on adenosine diphosphate glucose pyrophosphorylase (ADP) induced platelet aggregation in vivo. They found that the effect of compound prescription group was obviously superior to Ligustrazine group, which provided reference for the further study of antiplatelet with traditional Chinese preparation.

Stem cell transplantation is also a hot issue. It may be a new dawn for the treatment of myocardial infarction, and its preliminary clinical study outcome was inspiring. A study [51] indicated that bone marrow stem cell could transversely transform into myocardial cell and vascular endothelial cell in the environmental conditions of the heart, which finally remedy the damaged myocardium. Another study showed that ginsenoside Rg1 could induce bone marrow cells' migration to myocardium and differentiation to vascular endothelial cell by stimulating local myocardium to excrete granulocyte colony stimulating factor (G-CSF) [52]. When applied Chinese compound prescription based on ginseng and *Salvia miltiorrhiza* combined with bone marrow mononuclear cells autotransplantation through cardiac catheter to the model of myocardial infarction in swine, it promoted transplanted cell to survive, differentiate, and amplify, and many new myocardial cell and myocardial small vessel emerged. These facilitated the repair of damaged myocardial cells by synergies and complementing each other's advantages [53] and predicted a gratifying future. In addition, whether Chinese medicine could work on the inflammation reaction and local microcirculation after stem cell transplantation and immunological rejection after cell transplantation and whether it could improve transplanted cell viability and induce differentiation of transplanted cell need to be answered in the future investigations.

## 7. Conclusion

In conclusion, integrative medical research on CHD has made great development. Present studies shed light on the orientation of integrative medicine. From the progress of integrative medicine on CHD, in order to further complement each other's advantages of Chinese and Western medicine, researches should keep focusing research on original innovation of TCM etiology and pathogenesis, combination of disease and TCM syndrome, biological basis of TCM syndrome of CHD, difficulties and hot issues of modern medicine such as stem cell transplantation and strengthen clinical design and quality control of integrative medicine research. In addition, herb-drug interaction should not be ignored.
